# Comparative and Expression Analysis of Ubiquitin Conjugating Domain-Containing Genes in Two *Pyrus* Species

**DOI:** 10.3390/cells7070077

**Published:** 2018-07-16

**Authors:** Yunpeng Cao, Dandan Meng, Yu Chen, Muhammad Abdullah, Qing Jin, Yi Lin, Yongping Cai

**Affiliations:** School of Life Sciences, Anhui Agricultural University, Hefei 230036, China; xfcypeng@126.com (Y.C.); mdd5749@163.com (D.M.); jacyhao@163.com (Y.C.) cyp@ahau.edu.cn (M.A.); qingjin@ahau.edu.cn (Q.J.); linyi320722@163.com (Y.L.)

**Keywords:** UBC, *Pyrus*, expression, gene pairs

## Abstract

Ripening affects the nutritional contents and quality of fleshy fruits, and it plays an important role during the process of fruit development. Studies have demonstrated that ubiquitin-conjugating (*UBC* or *E2*) genes can regulate fruit ripening, but the characterization of *UBCs* in pear is not well documented. The recently published genome-wide sequences of *Pyrus bretschneideri* and *Pyrus communis* have allowed a comprehensive analysis of this important gene family in pear. Using bioinformatics approaches, we identified 83 (*PbrUBCs*) and 84 (*PcpUBCs*) genes from *P. bretschneideri* and *P. communis*, respectively, which were divided into 13 subfamilies. In total, 198 *PbrUBC* paralogous, 215 *PcpUBC* paralogous, and 129 orthologous gene pairs were detected. Some paralogous gene pairs were found to be distributed on the same chromosome, suggesting that these paralogs may be caused by tandem duplications. The expression patterns of most *UBC* genes were divergent between *Pyrus bretschneideri* and *Pyrus communis* during pear fruit development. Remarkably, the transcriptome data showed that *UBC* genes might play a more important role in fruit ripening for further study. This is the first report on the systematic analysis of two *Pyrus UBC* gene families, and these data will help further study the role of *UBC* genes in fruit development and ripening, as well as contribute to the functional verification of *UBC* genes in pear.

## 1. Introduction

Ubiquitination is an essential cellular process for eukaryotes [1]. The ubiquitin proteasome pathway involves many aspects of eukaryotic cell regulation because of its ability to degrade intracellular proteins [2,3]. Ubiquitin conjugation is a multistep reaction mediated by the action of three enzymes, including E1s (ubiquitin-activating enzymes), E2s (ubiquitin-conjugating enzymes), and E3s (ubiquitin ligases). E2s act in the middle step of the protein ubiquitination pathway [4]. Previous reports suggested that the E2 family members have a certain degree of expansion during evolution, for example, more ancestral eukaryotes such as algae have fewer E2 enzymes (< or =20) than certain plants and animals (>40) [5]. The *Saccharomyces cerevisiae* genome encodes 13 UBC proteins [6]; 19, 18, and 12 UBC proteins are identified in the algae *Chlamydomonas reinhardtii*, *Micromonas* sp. *RCC299*, and *Ostreococcus*, respectively; 20 in *Caenorhabditis elegans* [7]; and 75, 74, 52, 48, 34, and 37 UBC proteins in *Zea mays*, *Musa nana*, *Solanum lycopersicum*, *Oryza sativa*, *Carica papaya*, and *A. thaliana*, respectively [1,8,9,10,11,12].

Researchers have studied *UBC* genes in some plants, indicating that these genes are involved in tolerance against biotic and abiotic stresses, and plant growth and development [1,8,9,10,11,12]. For example, the *A. thaliana UBC1* and *AtUBC2* participate in the activation of the flowering suppressor *FLC* gene and inhibit flowering [13]. Overexpression of the *AtUBC32* gene in *A. thaliana* reduced the sensitivity of plants to salt stress [14]. Overexpression of *Vigna radiata UBC1* (*VrUBC1*), *Arachis hypogaea UBC2* (*AhUBC2*), or *Glycine max UBC2* (*GmUBC2*) in *A. thaliana* can enhance plant drought resistance [15,16,17]. The *A. thaliana UBC13* (*AtUBC13*) has been implicated in iron deficiency responses and epidermal cell differentiation [18,19]. Additionally, *AtUBC21* (*AtPEX4*) has been shown to be specific for ubiquitination in peroxisome maintenance [20]. In *Z. mays*, 16 and 48 *ZmUBCs* were significantly up-regulated in response to drought and salt stress [10], respectively. Similarly, in *O. sativa*, 14 *OsUBC* genes were differentially expressed under salt and drought stress [21].

Pear (*Pyrus* spp.) is one of the leading cultivated fruit trees which are widely grown in temperate regions, and its fleshy fruits play an important role in human health and nutrition [22,23]. The pear is the third largest temperate fruit tree after apple (*Malus domestica*) and grape (*Vitis vinifera*). Previously published manuscripts have carried out some studies on the mechanisms related to fruit ripening, as the fruit ripening is an important and complex process. The *UBC* genes play an important role in the fruit ripening process. In *S. lycopersicum*, Wang et al. (2014) found that *SlUBC32* was down-regulated in the *S. lycopersicum* rin mutant and up-regulated during fruit ripening, indicating that this gene plays a key role in the regulation of fruit ripening [11]. In *M. nana*, Dong et al. (2016) identified that the expressions of 32 *MaUBCs* were increased or decreased during different ripening stages [9]. In *C. papaya*, Jue et al. (2017) suggested that 13 and two *CpUBC*s were up-regulated and down-regulated during one and two ripening stages, respectively [12]. However, the *UBC* genes involved in fruit ripening in two *Pyrus* species (*P. bretschneideri* and *P. communis*) have not yet been identified. To further understand the function of *UBC* genes, we carried out a systematic analysis of two *Pyrus* species, including phylogenetic relationships, sequence characteristics, chromosomal locations, and the expression differences during fruit development. These results highlight the role of *UBC* genes in pear fruit development and provide important information for further exploring the functional differences between two *Pyrus* species.

## 2. Materials and Methods

### 2.1. Sequence Retrieval and Identification of UBC Genes

To identify the UBC proteins in two *Pyrus* species (i.e., *P. bretschneideri* and *P. communis*), we used HMMER v3.1b2 obtained from the HMMER website (http://www.hmmer.org/download.html) [24]. The HMM profile of the UBC domain (PF00179) was downloaded from Pfam 31.0 (http://pfam.xfam.org/) [25]. Using HMMER v3.1b2 software, an HMM search was carried out for *P. bretschneideri* [23] and *P. communis* [26] genomes with a significance *e*-value of 0.001. SMART [27], Pfam [25], and INTERPRO [28] were used to confirm the presence of the UBC domain. Information on *PbrUBCs* and *PcpUBCs*, including intron and exon numbers, chromosomal locations, and coding sequences (CDS), was obtained from the GigdDB (http://gigadb.org/) and GDR databases (https://www.rosaceae.org/) [29], respectively.

### 2.2. Phylogenetic Analysis and Gene Duplication

The MUSCLE program was used to perform the alignments of all UBC amino acid sequences using default parameters [30]. ModelFinder was used to detect the best substitution model of these alignment sequences. The phylogenetic tree was generated using full-length sequences by the Maximum Likelihood (ML) method with 1000 bootstrap replications and the VT + G4 model implemented in IQ-TREE software obtained from IQ-TREE (http://www.iqtree.org/) [31]. The FigTree software (http://tree.bio.ed.ac.uk/software/figtree/) was used to visualize the ML tree. We have utilized the MCscanX software for the analysis of gene duplication events [32], and the “add_ka_and_ks_to_collinearity.pl” script for the analysis of the non-synonymous (Ka)/synonymous (Ks) substitution ratio.

### 2.3. Gene Structure and Motif Analysis

The GFF files of *P. bretschneideri* and *P. communis* were downloaded from GigdDB (http://gigadb.org/) and GDR databases (https://www.rosaceae.org/), respectively. The TBtools software was used to plot the map of the exon-intron structure [33]. The MEME online tool was used to search for the conservative motif of both PbrUBC and PcpUBC proteins, with the maximum width of 200 amino acids and a limit of 20 motifs, and other default parameters [34].

### 2.4. Expression Analysis

To further understand the expression of *UBC* genes in both *P. bretschneideri* and *P. communis*, we downloaded the RNA-Seq data from the public NCBI database. The sample details and accession numbers for the above data are presented in the availability of data and materials section. The FASTX-toolkit was used to remove the low-quality base-calls (Q < 20) of raw reads [35]. The TopHat2 software was used to map the clean reads to the reference genome with default parameters [36], and the Cufflinks software was used to assemble and calculate the expression FPKM (Fragments Per Kilobase of exon model per Million mapped fragments) values [37]. The R software was used to plot the heatmap of these *UBC* genes.

### 2.5. Expression Correlation of Orthologous UBC Genes in Two Pyrus Species

Using RNA-Seq data, we obtained the expression profiles of orthologous *UBC* genes. Then, we estimated the similarity between the expression patterns of the orthologous gene pair by using Pearson’s correlation coefficient (*r*). The degree of expression diversity was confirmed by significant values (*r*) based on previous studies [38,39]. In general, *r* > 0.5, 0.3 < *r* < 0.5, and *r* < 0.3 suggest non-divergence, ongoing divergent, and divergent, respectively [38,39].

### 2.6. Availability of Data and Materials

*P. bretschneideri* fruit developmental stage 1 (Fruit_stage1: 15DAB), Accession: SRX1595645; *P. bretschneideri* Fruit_stage2 (30DAB), Accession: SRX1595646; *P. bretschneideri* Fruit_stage3 (55DAB), Accession: SRX1595647; *P. bretschneideri* Fruit_stage4 (85 DAB), Accession: SRX1595648; *P. bretschneideri* Fruit_stage5 (115 DAB), Accession: SRX1595650; *P. bretschneideri* Fruit_stage6 (mature stage), Accession: SRX1595651; *P. bretschneideri* Fruit_stage7 (fruit senescence stage), Accession: SRX1595652; *P. communis* Fruit_stage1 (15DAB), Accession: SRX1595636; *P. communis* Fruit_stage2 (30DAB), Accession: SRX1595637; *P. communis* Fruit_stage3 (55DAB), Accession: SRX1595638; *P. communis* Fruit_stage4 (85 DAB), Accession: SRX1595639; *P. communis* Fruit_stage5 (115 DAB), Accession: SRX1595640; *P. communis* Fruit_stage6 (mature stage), Accession: SRX1595641; *P. communis* Fruit_stage7 (fruit senescence stage), Accession: SRX1595644. *P. bretschneideri* drought-tolerant for 0 h, Accession: SRX4110141; *P. bretschneideri* drought-tolerant for 1 h, Accession: SRX4110140; *P. bretschneideri* drought-tolerant for 3 h, Accession: SRX4110143; *P. bretschneideri* drought-tolerant for 6 h, Accession: SRX4110142; *P. bretschneideri* recovery for 24 h, Accession: SRX4110139.

## 3. Results

### 3.1. Identification of UBC Genes in Two Pyrus Species

The protein of *P. bretschneideri* and *P. communis* was downloaded from the GigdDB (http://gigadb.org/) and GDR database (https://www.rosaceae.org/), respectively. To identify the potential UBC protein family in two *Pyrus* species, we obtained the UBC domain from the Pfam database and generated the HMM profile in the HMMER 3.0 package. A total of 85 and 94 putative UBC proteins were identified by searching the generated HMM profile with the E-value of 0.001 against the *P. bretschneideri* and *P. communis* protein sequence database, respectively. Further scanning of these UBC proteins for the UBC domain was conducted by a motif scan using the INTERPRO, SMART, and Pfam database, and found some UBC proteins not contained in the UBC domain. Finally, we identified 83 and 84 putative UBC proteins in the *P. bretschneideri* and *P. communis* genome, named PbrUBC01-83 and PcpUBC01-84 according to their order on the chromosomes, respectively. The information of these *PbrUBC* and *PcpUBC* genes, such as chromosome location, gene identifier, and protein length (aa), is shown in Table 1.

### 3.2. Phylogenetic Analysis of UBC Genes in Two Pyrus Species

**Figure 1:**
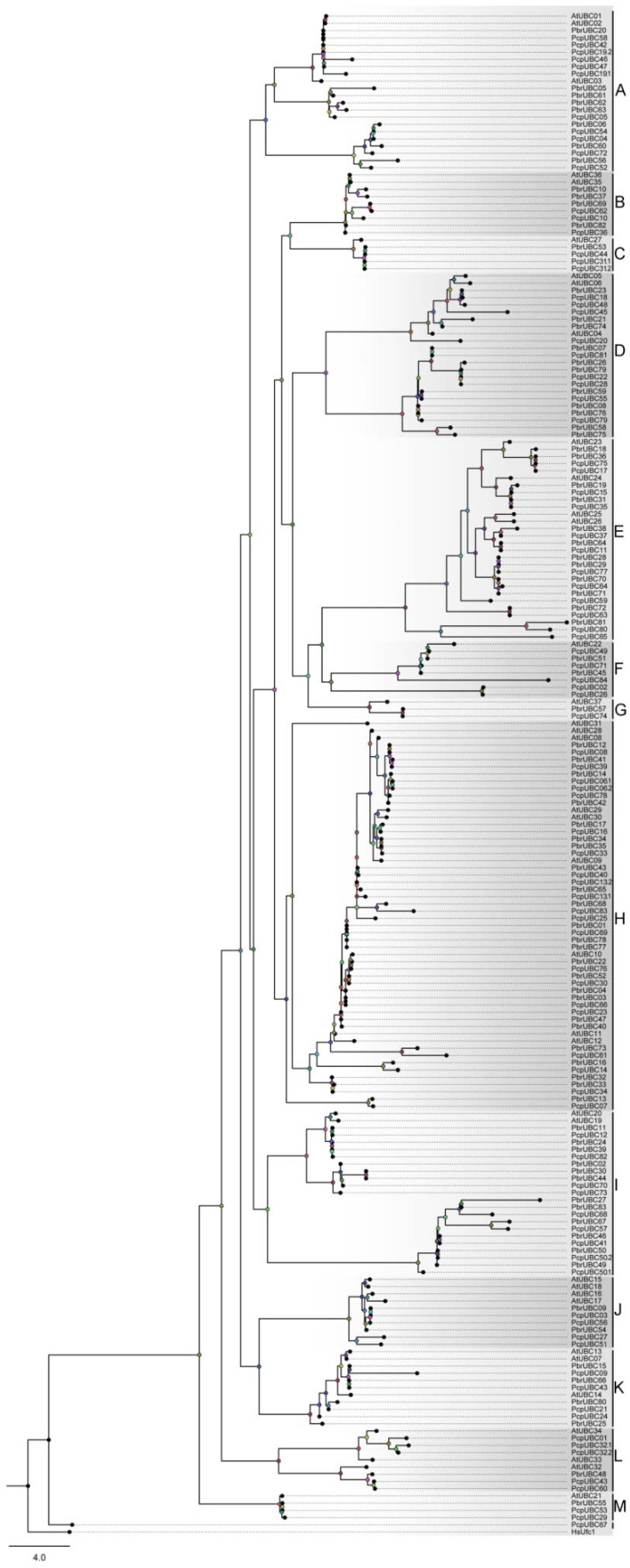
Phylogenetic tree of *UBC* genes from *P. bretschneideri*, *P. communis*, and *A. thaliana*. The phylogenetic tree was built using IQ-TREE software, and *HsUfc1* from *Homo sapiens* was used as the out-group. According to published articles, the ML tree could be divided into 13 subfamilies (A–M).

**Figure 2:**
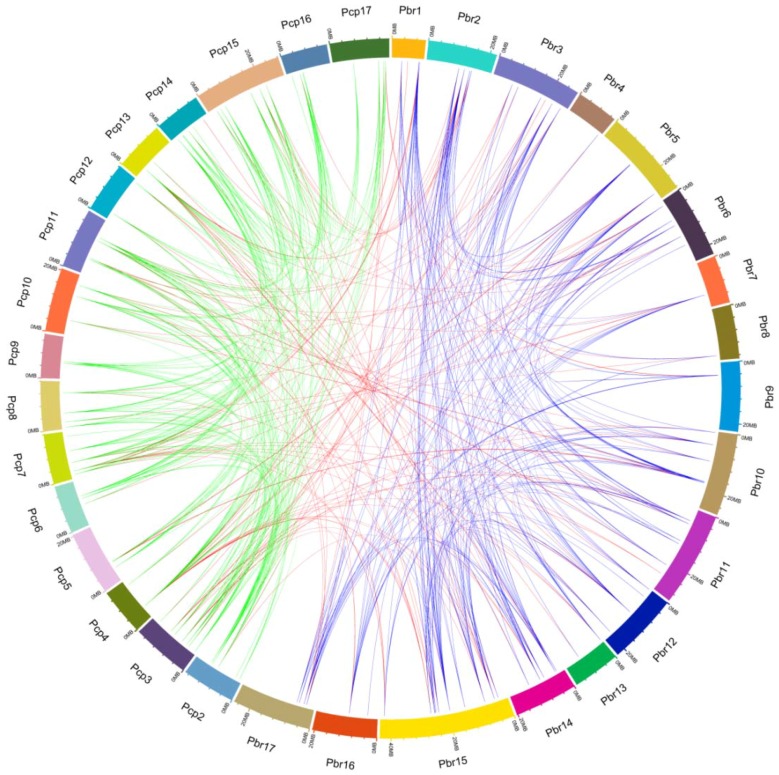
Microsynteny of *UBC* genes across *P. bretschneideri* and *P. communis*. The outermost scale represents the megabases (Mb). The *P. communis* and *P. bretschneideri* chromosomes are labeled Pcp and Pbr, and are represented by different color boxes, respectively. Blue, green, and red lines represent the *P. bretschneideri* paralogous gene pairs, *P. communis* paralogous gene pairs, and orthologous gene pairs, respectively.

To gain insight into the evolutionary relationships of *UBC* genes in two *Pyrus* species, we built an ML tree with all *PbrUBCs* and *PcpUBCs* sequences using IQ-TREE software with the VT+G4 model, and investigated the gene structures of *PbrUBC* and *PcpUBC* genes based on the GFF3 annotation files. Phylogenetic analysis revealed that these *PbrUBCs* and *PcpUBCs* could be clustered into 13 subfamilies (Figure 1), using *A. thaliana UBC* genes as a template [1]. Subfamily H had 45 *Pyrus UBC* members and was the largest clade of all subfamilies, which represented 26.01% of the total *Pyrus UBC* genes. However, subfamily M and subfamily G only contained three and two *Pyrus UBC* members, respectively. We also found that the distribution of *Pyrus UBC* members was uneven in some subfamilies, suggesting that they had undergone dynamic changes from the common ancestor. Based on the phylogenetic analysis, we found that the *UBC* members from these *Pyrus* species presented a higher similarity with each other, which was consistent with their (i.e., *P. bretschneideri* and *P. communis*) evolutionary relationship. Additionally, we also detected the orthologous gene pairs between *P. bretschneideri* and *P. communis*. Finally, 129 orthologous gene pairs were found in these *Pyrus* species (Figure 2 and Table S1). This orthologous analysis supported the evolutionary relationships and the classification of subfamilies of *UBC* genes between the *P. bretschneideri* and *P. communis* genome.

Observation of the gene structure in these two *Pyrus* species *UBC* genes showed that the numbers of introns in the 83 *PbrUBC* and 84 *PcpUBC* genes varied from 0 (*PbrUBC44*, *PcpUBC34*, *PbrUBC33*, *PcpUBC14*, *PbrUBC32*, *PbrUBC72*, *PcpUBC02*, *PcpUBC34*, *PcpUBC59*, and *PcpUBC63*) to 16 (*PcpUBC29*) (Figure S1). Additionally, we found that most of the *Pyrus UBC* genes clustered in the same subfamily contained highly similar gene structure maps, including intron numbers and exon length. For instance, *PbrUBC53* and *PcpUBC44* in the subfamily C contained four introns, and *PbrUBC10* and *PbrUBC37* in the subfamily B had five introns. We also scanned the conserved motifs in these *UBC* genes, and found that motif 2, −3, and −15 encoded the UBC domain (Figure S2). To sum up, the gene structures and conserved motifs of *UBC* genes were basically consistent with the above evolutionary relationship.

In general, the different protein isoforms produced by alternative splicing may affect the diversity of transcriptomics and proteomics, ultimately affecting gene expression regulation and protein function. In our study, the occurrence of alternative splicing events was revealed in the *UBC* family during evolution, such as *PcpUBC06*, *PcpUBC13*, *PcpUBC19*, *PcpUBC31*, *PcpUBC32*, and *PcpUBC50* (Table 1). The mRNAs of *PcpUBC13.1*/*PcpUBC13.2*, *PcpUBC19.1*/*PcpUBC19.2*, *PcpUBC32.1*/*PcpUBC32.2*, and *PcpUBC50.1*/*PcpUBC50.2*, which are produced by variable splicing, are different in the 3′-end. However, the mRNAs of *PcpUBC06.1*/*PcpUBC06.2* and *PcpUBC31.1*/*PcpUBC31.2*, which are produced by variable splicing, are different in the 5′-end (Figure S3). These results suggested that changes in the transcript sequence of the *UBC* gene caused by alternative splicing events may have an effect on the interaction ability and function of the encoded proteins.

### 3.3. Chromosomal Distribution and Gene Duplication of UBC Genes in Two Pyrus Species

**Figure 3:**
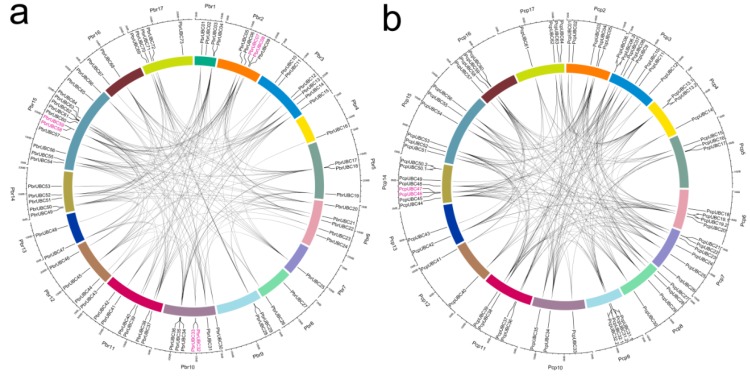
Localization and duplication of *UBC* genes in the *P. bretschneideri* (**a**) and *P. communis* (**b**) genome, respectively. The localizations of *PbrUBCs* and *PcpUBCs* mapped on the *P. bretschneideri* and *P. communis* genome, respectively, were obtained from Circos software [40]. The *P. communis* and *P. bretschneideri* chromosomes are labeled Pcp and Pbr, and are represented by different color boxes, respectively. Red regions indicate tandem duplication, and grey lines represent segment duplication. The outermost scale represents the megabases (Mb).

In the present study, 167 genes were identified as members of the *UBC* gene family, with 83 *PbrUBC* genes in *P. bretschneideri* and 84 *PcpUBC* genes in *P. communis* (Table 1). Then, we determined the chromosomal distribution of each *UBC* gene. As shown in Figure 3 and Table 1, the distribution of 167 *UBC* genes on the chromosome is random, and some of them were located on scaffolds. The genome maps of the *UBC* genes suggested that *PbrUBC* genes were dispersed across all chromosomes; however, *PcpUBC* genes were mainly found on 16 out of 17 chromosomes, except for chromosome 1. In the *P. bretschneideri* genome, chromosome 15 had the maximum number of *PbrUBC* genes (13), while chromosome 7 contained only one gene (*PbrUBC15*) gene. In the *P. communis* genome, both chromosome 3 and 14 contained the most *PcpUBC* genes, followed by chromosome 7 (6) and 15 (6) (Figure 3).

Gene duplication contributes to the expansion of gene family members and diversification of protein functions. In general, if two genes are collinear, they are considered to have evolved from a duplication event. In order to further investigate the expansion mechanism of *UBC* gene family members, the occurrence of segmental duplication and tandem duplication events were analyzed during the evolution of this gene family. Finally, 198 and 215 duplication events (Figure 3) of the *P. bretschneideri* and *P. communis UBC* genes were identified, respectively. Among these duplication gene pairs, four and one gene pairs were identified to have evolved from tandem duplications in *P. bretschneideri* and *P. communis*, respectively, and the remaining gene pairs were involved in segmental duplications. Additionally, a series of several-for-one duplication events in *P. bretschneideri* and *P. communis UBC* genes were observed, such as *PbrUBC07*/*PbrUBC26*, *PbrUBC07*/*PbrUBC20*, *PcpUBC12*/*PcpUBC34*, and *PcpUBC12*/*PcpUBC48*, and it is envisaged that these genes may contribute to the expansion of *UBC* gene family members during evolution. The pear genome shared two whole-genome duplication (WGD) events, the ancient WGD occurred in ~140 MYA (Millions of years ago) (Ks ~ 1.5–1.8) and the recent WGD occurred in 30–45 MYA (Ks ~ 0.15–0.3). Subsequently, 15 and 14 duplication gene pairs (Table S2) were identified as being derived from ancient WGDs and recent WGDs in the *P. communis* genome, respectively. In the *P. bretschneideri* genome, 17 duplication gene pairs were evolved from the recent WGDs, and 13 from the ancient WGDs. These results suggested that two WGDs contribute to the expansion of *UBC* gene family members in the *Pyrus* genome.

### 3.4. Evolutionary Patterns in Two Pyrus Species

**Figure 4:**
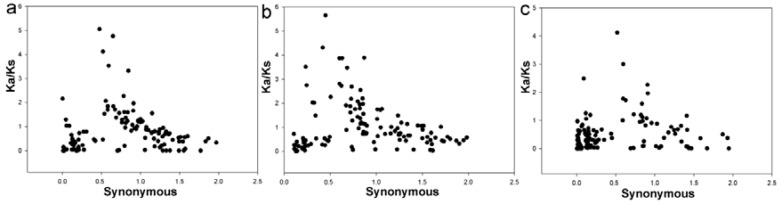
The distribution of Ka (nonsynonymous), Ks (synonymous), and Ka/Ks values of paralogous and orthologous gene pairs. (**a**–**c**) represent Pbr-Pbr, Pcp-Pcp, and Pbr-Pcp gene pairs, respectively. The *X*- and *Y*-axes denote the synonymous distance and Ka/Ks ratio for each pair, respectively.

To investigate the evolutionary divergence and patterns of the *UBC* genes in *P. bretschneideri* and *P. communis*, the selection pressures of 198 paralogous gene pairs in *P. bretschneideri*, 215 paralogous gene pairs in *P. communis*, and 129 orthologous gene pairs in *P. bretschneideri* and *P. communis* were analyzed. All gene pairs, including paralogous and orthologous, are listed in Table S1. To avoid the risk of saturation [41], we removed any Ks values >2.0 in our study. In *P. bretschneideri*, 99 paralogous pairs contained Ka/Ks ratios below one, while the remaining gene pairs had ratios greater than one (Table S2). In *P. communis*, 94 paralogous pairs had Ka/Ks ratios below one, while the remaining gene pairs had ratios greater than one. The maximum Ka/Ks value was 5.055 in *P. bretschneideri* (*PbrUBC04*-*PbrUBC70*) and 5.65 in *P. communis* (*PcpUBC51*-*PcpUBC58*) (Table S1 and Figure 4). Among these orthologous pairs, we found that the most of the gene pairs had Ka/Ks ratios that were below one, indicating that these genes (which evolved from a common ancestor) have undergone purify selection with slow evolution at the protein level. Remarkably, these genes might also evolve through positive selection (Ks = 0; Ka ≠ 0, such as *PbrUBC32*-*PcpUBC32*), negative selection (i.e., Ka = 0; Ks ≠ 0, such as *PcpUBC42*-*PcpUBC58*), and strongly negative selection (i.e., Ka = Ks = 0, such as *PbrUBC09*-*PcpUBC03*) due to these gene pairs being subject to strong constraints (Table S2).

### 3.5. Expression Profiles of UBC Genes in Pyrus Fruit Development

**Figure 5:**
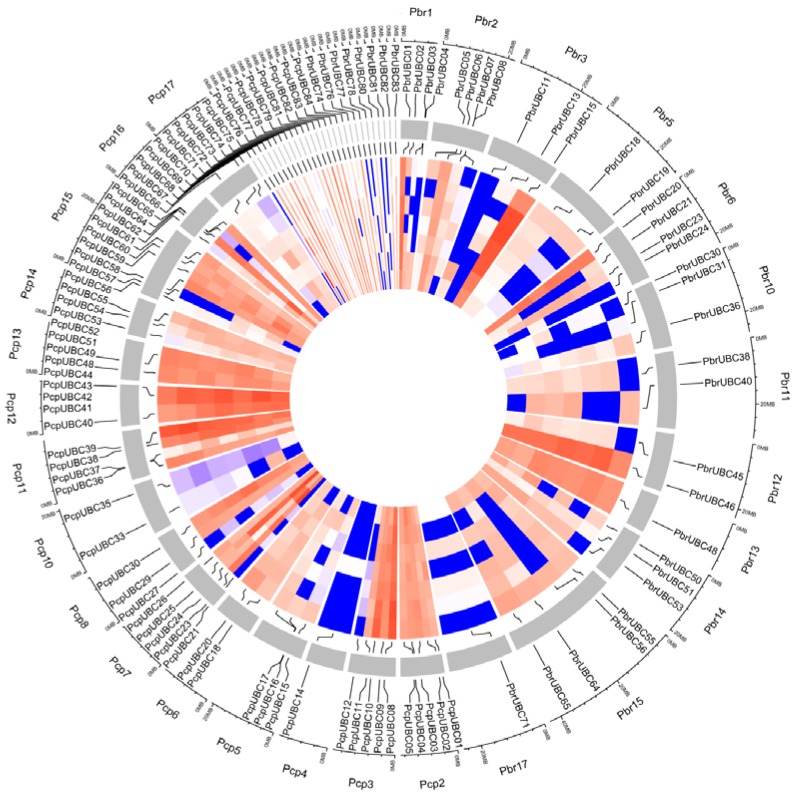
Expression of *UBC* genes from *P. bretschneideri* and *P. communis* during fruit development and ripening, including Fruit_stage1 (15 days after full blooming (DAB)), Fruit_stage2 (30 DAB), Fruit_stage3 (55 DAB), Fruit_stage4 (85 DAB), Fruit_stage5 (115 DAB), Fruit_stage6 (mature stage), and Fruit_stage7 (fruit senescence stage). The color scale represents normalized log 2-transformed, where grey indicates a medium level, blue indicates a low level, and red indicates a high level. Circos software was used to visualize the heat map. The FPKM values of *PbrUBCs* and *PcpUBCs* are presented in Table S3. The outermost ring represents Fruit_stage7, followed by Fruit_stage6, Fruit_stage5, Fruit_stage4, Fruit_stage3, and Fruit_stage2, and the innermost ring represents Fruit_stage1.

The genome sequences of both *P. bretschneideri* and *P. communis* provided an excellent opportunity to further study gene expression. Previous studies have shown that *UBC* genes may play an important role during fruit development [11,12]. To further understand the potential roles of *PbrUBC* and *PcpUBC* genes during pear fruit development, we obtained the transcriptome data of these *UBC* genes and built a heat map. From the transcriptome data results, it was apparent that 50.6% (42/83) *PbrUBCs* and 25% (21/84) *PcpUBCs* were not detected in each fruit developmental stage, suggesting their activity in other organs, such as the flower, root, or leaf. In *P. bretschneideri*, 41 *PbrUBC* genes were expressed in one or more developmental stages (Figure 5 and Table S2). Among them, 17 *PbrUBC* genes were expressed in all *P. bretschneideri* fruit development stages, indicating that these genes might be very important for the development and maturation of fruit. In *P. communis*, 47 *PcpUBC* genes were expressed in all *P. communis* fruit development stages, implying that these have functional activity in all fruit development stages. Remarkably, we found that the different isoforms produced by alternative splicing were not expressed in the period of pear fruit development, suggesting that the alternative splicing events might not play a role during pear fruit development. Additionally, we found that some *UBC* genes continuously increased or reduced at one or several stages, such as *PbrUBC24* and *PbrUBC80*, which were highly expressed in Fruit_stage3 (55 days after full blooming), and *PcpUBC14*, which was highly expressed at Fruit_stage5 (115 days after full blooming), implying that these genes might be very important for fruit-specific developmental stages.

### 3.6. Comparison of the Expression Patterns of UBC Genes in Two Pyrus Species

Pear is one of the leading cultivated fruit trees of temperate regions, and the fruit is the focus of this study due to its economic value. Homologous genes may have gene functional redundancy or divergence during evolution [42]. In the present study, to gain insight into the degree of expression diversity of *UBC* gene family members between *P. bretschneideri* and *P. communis*, their expression correlations were estimated using Pearson’s correlation coefficient (*r*). Remarkably, we only considered the homologous genes which were expressed in at least one pear fruit development stage (Table S4). Twenty-two orthologous gene pairs (such as *PbrUBC36-PcpUBC17*, *PbrUBC20-PcpUBC42*, and *PbrUBC06-PcpUBC54*) were found to be non-divergent, five orthologous gene pairs (such as *PbrUBC18-PcpUBC17*, *PbrUBC01-PcpUBC69*, *PbrUBC31-PcpUBC35*, *PbrUBC07-PcpUBC55*, and *PbrUBC80-PcpUBC21*) were ongoing divergent, and the remaining orthologous gene pairs (such as *PbrUBC50-PcpUBC53*, *PbrUBC19-PcpUBC35*, *PbrUBC04-PcpUBC21*, and *PbrUBC20-PcpUBC58*) were divergent (Table S3). These results suggested that most of the *UBC* orthologous gene pairs have undergone functional divergence.

### 3.7. Expression Profiles of PbrUBC Genes Respond to Drought Stress

**Figure 6:**
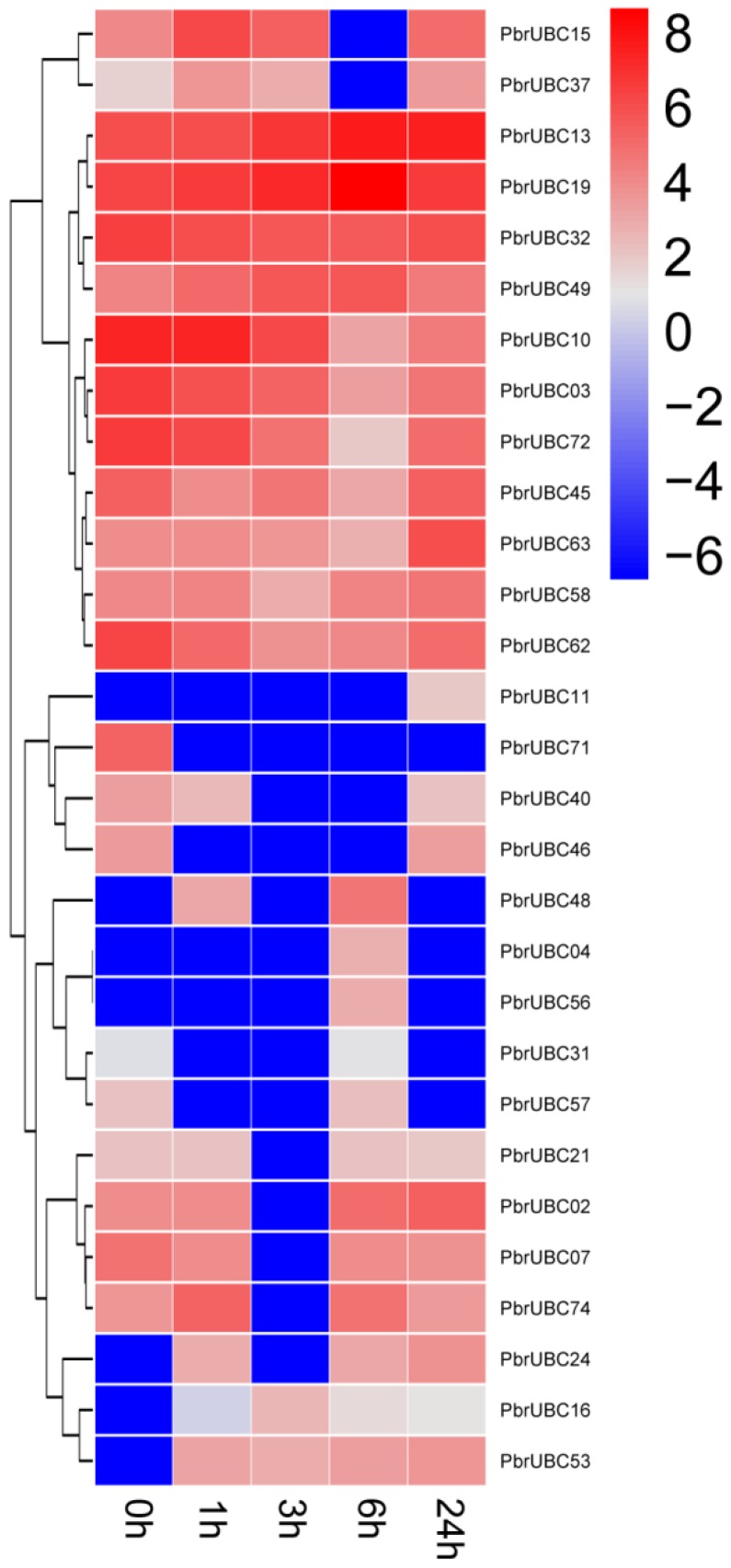
Expression analysis of *PbrUBC* genes under drought stress treatment. The color scale represents normalized log 2-transformed, where grey indicates a medium level, blue indicates a low level, and red indicates a high level. The treatments were indicated at the bottom of each column, and the genes are located on the right.

As a major abiotic stress, drought can affect plant productivity, growth, and development. Previous studies have shown that plants can enhance their drought tolerance by regulating gene transcription [9,15,16]. To identify *UBC* genes with a potential role in the drought stress response of *P. bretschneideri*, we carried out the expression analysis for 83 *PbrUBC* genes under drought stress. From the transcriptome data results, we found that only 34.9% (28/83) *PbrUBCs* were expressed under drought stress (Figure 6). Under drought stress treatment, five *PbrUBC* genes (*PbrUBC02*, *PbrUBC10*, *PbrUBC15*, *PbrUBC37*, and *PbrUBC74*) were up-regulated at early time points; however, they were down-regulated after a long period of stress treatment, indicating the existence of a possible feedback regulatory mechanism. Two (*PbrUBC49* and *PbrUBC63*) and five *PbrUBC* (*PbrUBC03*, *PbrUBC07*, *PbrUBC13*, *PbrUBC32*, and *PbrUBC62*) genes under drought stress treatment were up- and down-regulated, respectively (Figure 6). Our data indicated that these genes might be important for drought stress responses and will help to select candidate genes for functional analysis under drought stress.

## 4. Discussion

As a part of the ubiquitin proteasome system, ubiquitin-conjugating enzymes have been proved to play an important role in plant growth and development [1,11,21]. Although members of the *UBC* gene family have potential functional significance, they are relatively few in higher plants. Pear is widely cultivated in temperate regions due to its high nutritional and economic value. For pears, the fruit is the focus of this study. Previous studies have shown that the *UBC* gene family plays a very important role in fruit development and ripening [11,12], but is still excluded in two *Pyrus* species (*P. communis* and *P. bretschneideri*).

In our study, 83 *PbrUBCs* and 84 *PcpUBCs* genes were identified from the *P. bretschneideri* and *P. communis* genome, respectively. The number of *PbrUBCs* and *PcpUBCs* is much larger than 75, 74, 52, 48, 48, and 34 *UBC* genes previously reported from *Zea mays*, *Musa nana*, *Solanum lycopersicum*, *Oryza sativa*, *Arabidopsis thaliana*, and *Carica papaya*, respectively [1,8,9,10,11,12]. The genome sizes of *Zea mays*, *Musa nana*, *Solanum lycopersicum*, *Oryza sativa*, *Arabidopsis thaliana*, and *Carica papaya* are ~2300, ~523, ~900, ~466, ~125, and ~372 Mb, respectively. Then, we found that the genome sizes of *S. lycopersicum* and *O. sativa* are 7.2 and 3.7 times larger than that of *A. thaliana*, respectively; however, the genomes of these species have a similar number of *UBCs*, including 52, 48, and 48, respectively. In addition, the genome size of *Z. mays* is 4.67 times larger than that of *Pyrus* species (i.e., *P. bretschneideri* and *P. communis*), but the genomes of both *P. bretschneideri* (83) and *P. communis* (84) have a larger number of *UBCs* compared to *Z. mays* (75) and other studied species. Therefore, we speculate that the difference in the number of *UBC* genes is not related to the size of the genome.

Alternatively, gene duplication events, including segmental and tandem duplication, play a significant role in the expansion of gene family members in the genome. Two WGD events, including recent WGD [23] and ancient WGD [43], were shared by both the *P. bretschneideri* and *P. communis* genome during evolution. In order to understand the contribution of gene duplication events to the expansion of *UBC* family members in two *Pyrus* species, we analyzed the expansion mechanism of both the *PbrUBC* and *PcpUBC* gene family. In the *P. bretschneideri* genome, 192 *PbrUBC* gene pairs were determined to be involved in segmental duplication events and four gene pairs were identified that were involved in tandem duplication events. Similarly, 211 and one *UBC* gene pairs were involved in segmental duplication and tandem duplication events in the *P. communis* genome, respectively. These data indicate that the common expansion mechanism of the *UBC* gene family is mainly segmental duplication events, which is shared by both *PbrUBCs* and *PcpUBCs*. Therefore, we can infer that the expansion of *UBC* gene family members may not completely depend on independent duplications of individual sequences, and it may also be the result of rearrangement events and segmental chromosome duplication. A growing number of studies have shown that segmental duplications play a major role in the expansion of the pear gene family, such as the *VQ*, *MYB*, *PRX*, *PHD*, and *WOX* gene families [22,42,44,45,46].

*UBC* genes have been demonstrated to play an important role in plant growth and development, and physiological processes. For instance, *OsUBC1* from *O. sativa* involves cellular responses to abiotic and biotic stresses [47], and the expression of five *A. thaliana UBC* genes (*AtUBC13*, *AtUBC17*, *AtUBC20*, *AtUBC26*, and *AtUBC31*) and three *O. sativa UBC* genes (*OsUBC2*, *OsUBC5*, and *OsUBC18*) is significantly down-regulated under drought and salt stress treatments; however, three *OsUBC* genes (*OsUBC13*, *OsUBC15*, and *OsUBC45*) are significantly up-regulated [21]. In the present study, we found that 34.9% (28/83) of *PbrUBCs* can respond to drought stress treatment at the transcriptional level, implying these genes play essential roles in responsive to drought stress in *P. bretschneideri*, such as *PbrUBC02*, *PbrUBC10*, *PbrUBC15*, *PbrUBC37*, and *PbrUBC74*, which were up-regulated at early time points. In the *O. sativa* and *Z. mays*, similar expression changes among *UBC* genes were also observed, including the expression of 34 *ZmUBC* genes that changed significantly and were up-regulated during early time points. These data indicated that these *UBC* genes might have important roles under drought stress treatment during *P. bretschneideri* development. The function of *UBC* genes in plant development and response stress has been well studied, but little is known about the role of protein ubiquitination in fruit development and ripening, except for *M. nana*, *S. lycopersicum*, and *C. papaya*. In *M. nana*, five *UBC* genes (*MaUBC1*, *MaUBC9*, *MaUBC70*, *MaUBC68*, and *MaUBC71*) presented about 10-fold to 40-fold higher expression levels at the fifth stage than at other stages of fruit ripening; however, seven other *UBC* genes (*MaUBC8*, *MaUBC16*, *MaUBC17*, *MaUBC33*, *MaUBC34*, *MaUBC56*, and *MaUBC61*) presented continuously increasing expression during all the fruit development stages [9]. In *S. lycopersicum*, six *UBC* genes (*SlUBC6*, *SlUBC8*, *SlUBC24*, *SlUBC32*, *SlUBC41*, and *SlUBC42*) were directly regulated by RIN, a fruit-ripening regulator [11]. In *C. papaya*, 13 (*CpUBC4*, *CpUBC6*, *CpUBC7*, *CpUBC8*, *CpUBC9*, *CpUBC11*, *CpUBC12*, *CpUBC14*, *CpUBC16*, *CpUBC19*, *CpUBC20*, *CpUBC28*, and *CpUBC34*) and two (*CpUBC2* and *CpUBC10*) were up-regulated and down-regulated during *C. papaya* fruit ripening stages, respectively [12]. Our results suggested that *PbrUBC82*, *PcpUBC10*, and *PcpUBC62*, orthologs of *MaUBC3* and *MaUBC8* and *AtUBC36*, respectively (Figure S4), contained high expression levels during the fruit developmental period. *S. lycopersicum SlUBC6* orthologs in two *Pyrus* species *PbrUBC18* and *PcpUBC17* were directly regulated by RIN (a fruit-ripening regulator), as reported in *S. lycopersicum*, and in two *Pyrus* species, were also highly expressed in fruits, suggesting a major role in fruit development. Additionally, we found that orthologs from different species exhibit different expression profiles. *SlUBC32*, *MaUBC72*, *MaUBC47*, *PcpUBC44*, *PbrUBC53*, *PcpUBC31*, and *CpUBC5*, which belong to the same subfamily (Figure S4), contained different expression profiles. For example, *MaUBC72* was expressed during all *M. nana* fruit development, while its *P. communis* and *S. lycopersicum* orthologous genes, *SlUBC32* and *PcpUBC44*/*-31*, were not expressed during fruit development. The contrary expression patterns suggested that these genes have different regulatory mechanisms in the development of plant fruits. Taken together, the present study indicated that some *PbrUBCs* and *PcpUBCs* might contribute to the regulation of fruit development and ripening processes. For *UBC* genes, the expression of most *UBC* orthologous gene pairs from *P. bretschneideri* and *P. communis* has undergone functional divergence, indicating functional redundancy evolved from a common ancestry for some orthologous gene pairs, and from neo-functionalization or sub-functionalization for others.

## Figures and Tables

**Table 1:** The detailed information of *UBC* family members in both *P. bretschneideri* and *P. communis.*

Gene Name	Gene Identifier	Chromosme	5′ End	3′ End	Protein Size (aa)
*PcpUBC01*	PCP011547.1	chr2	908379	915288	378
*PcpUBC02*	PCP008190.1	chr2	2303235	2304455	406
*PcpUBC03*	PCP026226.1	chr2	9163625	9165622	161
*PcpUBC04*	PCP025062.1	chr2	11398030	11400462	160
*PcpUBC05*	PCP029820.1	chr2	11983749	11985588	154
*PcpUBC06.1*	PCP020092.1	chr3	547762	556678	986
*PcpUBC06.2*	PCP041697.1	chr3	551276	556678	387
*PcpUBC07*	PCP000885.1	chr3	3236140	3247151	841
*PcpUBC08*	PCP013747.1	chr3	4266095	4267481	148
*PcpUBC09*	PCP031109.1	chr3	6092388	6096394	192
*PcpUBC10*	PCP029016.1	chr3	9480497	9483572	146
*PcpUBC11*	PCP029037.1	chr3	11156660	11161769	769
*PcpUBC12*	PCP008664.1	chr3	16102272	16105426	277
*PcpUBC13.1*	PCP006596.1	chr4	3944944	3952328	304
*PcpUBC13.2*	PCP033076.1	chr4	3944944	3946278	148
*PcpUBC14*	PCP032557.1	chr4	13065481	13065759	92
*PcpUBC15*	PCP004584.1	chr5	4531710	4535205	920
*PcpUBC16*	PCP000343.1	chr5	6602829	6603942	120
*PcpUBC17*	PCP000325.1	chr5	6785831	6791593	1148
*PcpUBC18*	PCP013561.1	chr6	7061585	7063890	183
*PcpUBC19.1*	PCP003994.1	chr6	9051182	9054610	490
*PcpUBC19.2*	PCP038680.1	chr6	9053326	9054610	152
*PcpUBC20*	PCP006677.1	chr6	10001882	10012807	890
*PcpUBC21*	PCP025848.1	chr7	1250213	1253437	172
*PcpUBC22*	PCP025852.1	chr7	1277835	1279055	189
*PcpUBC23*	PCP027463.1	chr7	2394123	2396438	148
*PcpUBC24*	PCP039252.1	chr7	5395929	5399115	181
*PcpUBC25*	PCP041398.1	chr7	9425098	9426267	78
*PcpUBC26*	PCP023269.1	chr7	14434633	14435892	419
*PcpUBC27*	PCP018282.1	chr8	526845	534958	439
*PcpUBC28*	PCP024977.1	chr8	2320747	2321922	189
*PcpUBC29*	PCP014622.1	chr8	5359408	5371794	989
*PcpUBC30*	PCP013024.1	chr8	12182295	12185615	188
*PcpUBC31.1*	PCP025013.1	chr9	5181126	5187890	423
*PcpUBC31.2*	PCP042586.1	chr9	5181126	5183531	195
*PcpUBC32.1*	PCP029211.1	chr9	5697502	5704326	356
*PcpUBC32.2*	PCP037499.1	chr9	5697502	5700526	237
*PcpUBC33*	PCP022975.1	chr10	4876099	4877045	137
*PcpUBC34*	PCP006043.1	chr10	13347113	13347565	150
*PcpUBC35*	PCP019803.1	chr10	17163601	17167148	921
*PcpUBC36*	PCP037747.1	chr11	5179080	5182742	153
*PcpUBC37*	PCP003934.1	chr11	5615653	5620782	686
*PcpUBC38*	PCP014945.1	chr11	12048835	12052442	227
*PcpUBC39*	PCP007359.1	chr11	13280818	13282231	148
*PcpUBC40*	PCP041407.1	chr12	2420398	2421551	148
*PcpUBC41*	PCP043134.1	chr12	15435377	15437609	146
*PcpUBC42*	PCP003304.1	chr13	3769096	3771160	152
*PcpUBC43*	PCP028885.1	chr13	7446155	7448170	373
*PcpUBC44*	PCP010847.1	chr14	1973506	1975549	195
*PcpUBC45*	PCP000493.1	chr14	3899314	3905194	754
*PcpUBC46*	PCP006717.1	chr14	4144989	4159895	1373
*PcpUBC47*	PCP033096.1	chr14	4155864	4157211	152
*PcpUBC48*	PCP017973.1	chr14	6488805	6490257	144
*PcpUBC49*	PCP031605.1	chr14	8511513	8512945	265
*PcpUBC50.1*	PCP001680.1	chr14	12856983	12862739	444
*PcpUBC50.2*	PCP032125.1	chr14	12860593	12862739	146
*PcpUBC51*	PCP022610.1	chr15	2571489	2577399	467
*PcpUBC52*	PCP032902.1	chr15	3995850	3996286	78
*PcpUBC53*	PCP013613.1	chr15	5801442	5811234	828
*PcpUBC54*	PCP005846.1	chr15	15546784	15549513	160
*PcpUBC55*	PCP020991.1	chr15	18866853	18872463	319
*PcpUBC56*	PCP014540.1	chr15	21362811	21364686	161
*PcpUBC57*	PCP006806.1	chr16	1486195	1494553	341
*PcpUBC58*	PCP026158.1	chr16	4290275	4292359	152
*PcpUBC59*	PCP021281.1	chr16	5293770	5294891	373
*PcpUBC60*	PCP021299.1	chr16	5419315	5421158	307
*PcpUBC61*	PCP010786.1	chr17	6829215	6832764	267
*PcpUBC62*	PCP042470.1	chr17	15393404	15402997	596
*PcpUBC63*	PCP012277.1	chr17	17062013	17063062	349
*PcpUBC64*	PCP007143.1	chr17	17728444	17733945	621
*PcpUBC65*	PCP018399.1	scaffold00612	73468	83219	651
*PcpUBC66*	PCP021641.1	scaffold00634	76924	79227	148
*PcpUBC67*	PCP007398.1	scaffold00805	133398	134716	174
*PcpUBC68*	PCP018521.1	scaffold00852	13601	20410	278
*PcpUBC69*	PCP004264.1	scaffold00983	16646	19316	148
*PcpUBC70*	PCP021954.1	scaffold01394	34467	36817	191
*PcpUBC71*	PCP007629.1	scaffold01465	20581	22904	291
*PcpUBC72*	PCP002761.1	scaffold01522	35381	36867	160
*PcpUBC73*	PCP020361.1	scaffold01593	68663	70923	191
*PcpUBC74*	PCP045045.1	scaffold01774	29298	33191	754
*PcpUBC75*	PCP001255.1	scaffold01881	39525	44855	1147
*PcpUBC76*	PCP004531.1	scaffold01923	15838	21766	337
*PcpUBC77*	PCP028469.1	scaffold02358	32352	36077	463
*PcpUBC78*	PCP022164.1	scaffold02454	7209	11865	389
*PcpUBC79*	PCP012568.1	scaffold02548	27443	29342	178
*PcpUBC80*	PCP043259.1	scaffold04878	3046	6910	134
*PcpUBC81*	PCP001444.1	scaffold05041	9798	11719	183
*PcpUBC82*	PCP022346.1	scaffold17014	829	2135	180
*PcpUBC83*	PCP040042.1	scaffold23907	470	1254	125
*PcpUBC84*	PCP011188.1	scaffold27287	92	1717	232
*PbrUBC01*	Pbr021045.1	Chr1	3279060	3282148	149
*PbrUBC02*	Pbr022046.1	Chr1	5355491	5357926	192
*PbrUBC03*	Pbr018716.1	Chr1	9129424	9132884	149
*PbrUBC04*	Pbr013632.1	Chr1	9364933	9367901	149
*PbrUBC05*	Pbr029889.2	Chr2	11205065	11207039	195
*PbrUBC06*	Pbr025178.1	Chr2	13123810	13126801	161
*PbrUBC07*	Pbr022865.1	Chr2	15059120	15062337	184
*PbrUBC08*	Pbr022866.1	Chr2	15070696	15073441	182
*PbrUBC09*	Pbr040498.1	Chr2	15603251	15605586	162
*PbrUBC10*	Pbr024232.2	Chr3	7098302	7101956	160
*PbrUBC11*	Pbr027637.1	Chr3	9231735	9233562	181
*PbrUBC12*	Pbr023139.1	Chr3	17780615	17782004	149
*PbrUBC13*	Pbr000740.1	Chr3	19467052	19469759	170
*PbrUBC14*	Pbr013150.2	Chr3	22108425	22111475	149
*PbrUBC15*	Pbr034016.1	Chr3	25287937	25291916	168
*PbrUBC16*	Pbr030934.3	Chr4	12531057	12533684	161
*PbrUBC17*	Pbr027417.1	Chr5	12936909	12946872	201
*PbrUBC18*	Pbr027395.1	Chr5	13135775	13142057	1149
*PbrUBC19*	Pbr000361.1	Chr5	25903450	25906904	853
*PbrUBC20*	Pbr011471.1	Chr6	1539205	1541448	153
*PbrUBC21*	Pbr009129.1	Chr6	7334609	7336195	77
*PbrUBC22*	Pbr014124.1	Chr6	9284322	9290606	296
*PbrUBC23*	Pbr018194.1	Chr6	13523851	13526524	184
*PbrUBC24*	Pbr040529.1	Chr6	16795923	16797553	181
*PbrUBC25*	Pbr032353.1	Chr7	10867139	10869902	224
*PbrUBC26*	Pbr006183.1	Chr8	15645575	15647047	190
*PbrUBC27*	Pbr004154.1	Chr8	4690292	4691740	129
*PbrUBC28*	Pbr032653.1	Chr9	4250106	4253822	461
*PbrUBC29*	Pbr032645.1	Chr9	4153561	4154900	279
*PbrUBC30*	Pbr031810.1	Chr10	268749	273571	458
*PbrUBC31*	Pbr016259.1	Chr10	4489977	4494846	922
*PbrUBC32*	Pbr009080.1	Chr10	10152737	10153189	151
*PbrUBC33*	Pbr009081.1	Chr10	10158759	10159211	151
*PbrUBC34*	Pbr020740.1	Chr10	17324391	17325467	149
*PbrUBC35*	Pbr020719.1	Chr10	17573570	17574635	149
*PbrUBC36*	Pbr020703.1	Chr10	17785964	17790523	891
*PbrUBC37*	Pbr038220.3	Chr11	4285552	4289283	190
*PbrUBC38*	Pbr038323.1	Chr11	5463176	5468616	589
*PbrUBC39*	Pbr017901.1	Chr11	11843306	11844937	181
*PbrUBC40*	Pbr031559.1	Chr11	13000377	13002724	149
*PbrUBC41*	Pbr041320.1	Chr11	21287679	21289091	152
*PbrUBC42*	Pbr017298.1	Chr11	24728725	24731067	149
*PbrUBC43*	Pbr028474.1	Chr12	194166	195789	149
*PbrUBC44*	Pbr016440.1	Chr12	3192332	3192601	90
*PbrUBC45*	Pbr039044.1	Chr12	10209302	10211625	309
*PbrUBC46*	Pbr015391.1	Chr12	19750784	19753479	147
*PbrUBC47*	Pbr010810.1	Chr13	289004	291284	149
*PbrUBC48*	Pbr011958.2	Chr13	9713060	9715379	231
*PbrUBC49*	Pbr010372.1	Chr14	2425473	2427613	147
*PbrUBC50*	Pbr010424.1	Chr14	2933078	2935217	147
*PbrUBC51*	Pbr038166.1	Chr14	7236972	7240490	273
*PbrUBC52*	Pbr026720.1	Chr14	8739052	8742374	169
*PbrUBC53*	Pbr027115.1	Chr14	12937009	12939516	196
*PbrUBC54*	Pbr005908.1	Chr15	2962026	2964448	162
*PbrUBC55*	Pbr009224.1	Chr15	4281248	4283960	158
*PbrUBC56*	Pbr019673.1	Chr15	7696112	7697156	100
*PbrUBC57*	Pbr016945.1	Chr15	14705287	14708021	524
*PbrUBC58*	Pbr017248.1	Chr15	19933740	19935641	141
*PbrUBC59*	Pbr017249.1	Chr15	19937558	19939328	182
*PbrUBC60*	Pbr015294.2	Chr15	23696990	23705032	371
*PbrUBC61*	Pbr024308.1	Chr15	24667752	24669854	153
*PbrUBC62*	Pbr024286.1	Chr15	24961413	24963994	190
*PbrUBC63*	Pbr024279.1	Chr15	25164896	25167005	178
*PbrUBC64*	Pbr017425.1	Chr15	26387149	26392677	702
*PbrUBC65*	Pbr040652.1	Chr15	36951735	36952300	112
*PbrUBC66*	Pbr020836.2	Chr15	42195161	42198738	228
*PbrUBC67*	Pbr012108.1	Chr16	3304638	3306350	202
*PbrUBC68*	Pbr013690.1	Chr16	9644295	9646021	188
*PbrUBC69*	Pbr022472.1	Chr17	2772442	2779627	468
*PbrUBC70*	Pbr026816.1	Chr17	3678331	3681687	454
*PbrUBC71*	Pbr034051.1	Chr17	5335721	5339074	454
*PbrUBC72*	Pbr008641.1	Chr17	6164722	6165771	350
*PbrUBC73*	Pbr040232.1	Chr17	20655869	20656618	106
*PbrUBC74*	Pbr003941.1	scaffold1182.0	19987	21731	175
*PbrUBC75*	Pbr005003.1	scaffold1241.0	889	1709	133
*PbrUBC76*	Pbr005004.1	scaffold1241.0	11548	13331	182
*PbrUBC77*	Pbr006049.1	scaffold1301.0	22029	24744	149
*PbrUBC78*	Pbr009005.1	scaffold1564.0	5159	7986	149
*PbrUBC79*	Pbr028213.1	scaffold467.0	324186	326069	190
*PbrUBC80*	Pbr028219.1	scaffold467.0	348935	352574	172
*PbrUBC81*	Pbr032413.2	scaffold581.0.1	30262	34773	126
*PbrUBC82*	Pbr034367.1	scaffold640.0	26565	30347	154
*PbrUBC83*	Pbr042566.1	scaffold992.0	77372	83394	147

Note: Red logo represents tandem duplication.

## Supplementary Materials

The following are available online at http://www.mdpi.com/2073-4409/7/7/77/s1, Figure S1: Structure of both *PbrUBC* and *PcpUBC* genes. UTR, exons, and introns are indicated by green, yellow, and grey, respectively. The length of *PbrUBCs* and *PcpUBCs* can be estimated using the scale at the bottom. Figure S2: Conserved protein motifs of both PbrUBC and PcpUBC proteins. All motifs were scanned by MEME software using the complete amino acid sequences of all *P. bretschneideri* and *P. communis* UBC proteins documented in Figure S2: Using the scale at the bottom, the length of UBC protein and motif can be estimated. Figure S3: Schematic depictions of the alternatively spliced *UBC* genes. The introns and exons are represented by thin lines and green rectangles, respectively. The length of the alternatively spliced *UBC* genes can be estimated using the scale at the bottom. Figure S4: Phylogenetic tree of the UBC proteins from *P. bretschneideri*, *P. communis*, *A. thaliana*, *M. nana*, C*. papaya*, and *S. lycopersicum*. Complete alignments of all UBC proteins from *P. bretschneideri*, *P. communis*, *M. acuminate*, *C. papaya*, *S. lycopersicum*, and *A. thaliana* were carried out using the MUSCLE program. The tree was constructed using the Maximum Likelihood (ML) method with IQ-TREE, and HsUfc1 from Homo sapiens was used as the out-group. Table S1. Homologous analyses of *UBC* genes in *P. bretschneideri* and *P. communis*. The homologous analyses of *UBC* genes in *P. bretschneideri* and *P. communis* were carried out using MCscanX software. Table S2: Ka/Ks analysis of between *UBC* gene pairs in *Pyrus* species. The “add_ka_and_ks_to_collinearity.pl” script of MCscanX software was used to detect the non-synonymous (Ka)/synonymous (Ks) substitution ratio. Table S3: The FPKM (fragments per kilobaseof exon per million fragments mapped) values of *PbrUBC* and *PcpUBC* genes during fruit development and ripening. Table S4: Divergence analysis of orthologous *UBC* gene pairs among *P. bretschneideri* and *P. communis*. The Pearson’s correlation coefficient (*r*) was used to estimate the similarity between the expression patterns of the orthologous gene pair. In general, *r* > 0.5, 0.3 < *r* < 0.5, and *r* < 0.3 suggest non-divergence, ongoing divergent, and divergent, respectively.
