# Supplementary figures and images for: Comparative and Expression Analysis of Ubiquitin Conjugating Domain-Containing Genes in Two Pyrus Species
Source: Cells. 2018 Jul 16;7(7):77. doi: 10.3390/cells7070077 (PMC6071128; doi:10.3390/cells7070077)

UTR  
CDS

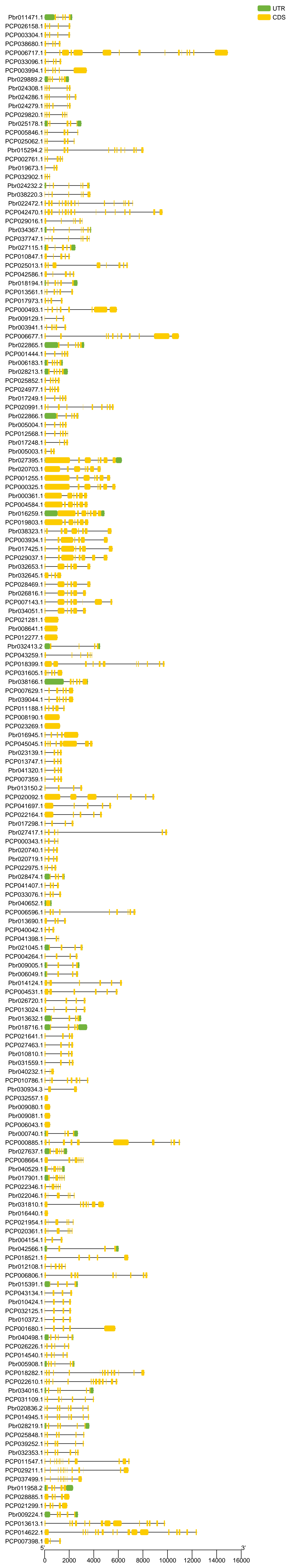

Supplement: Supplementary file 1 [file cells-07-00077-s001.zip › Figure S1.pdf]

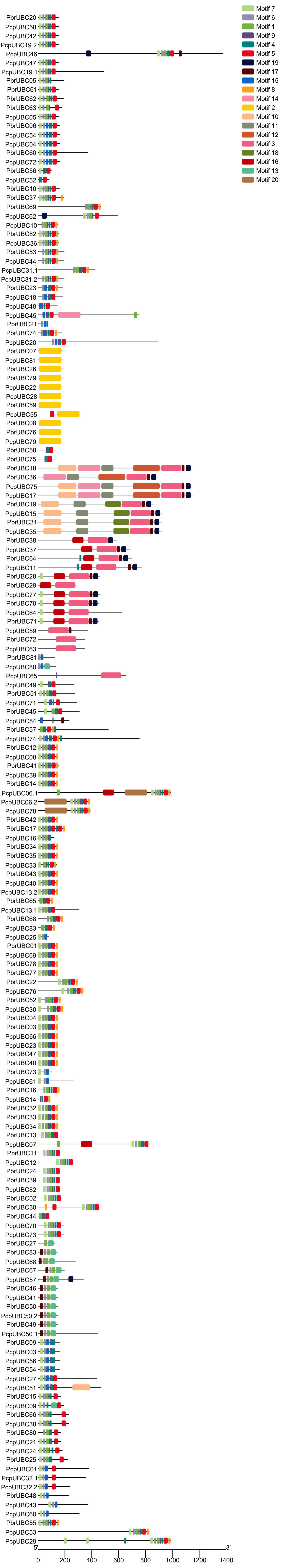

Supplement: Supplementary file 1 [file cells-07-00077-s001.zip › Figure S2.pdf]

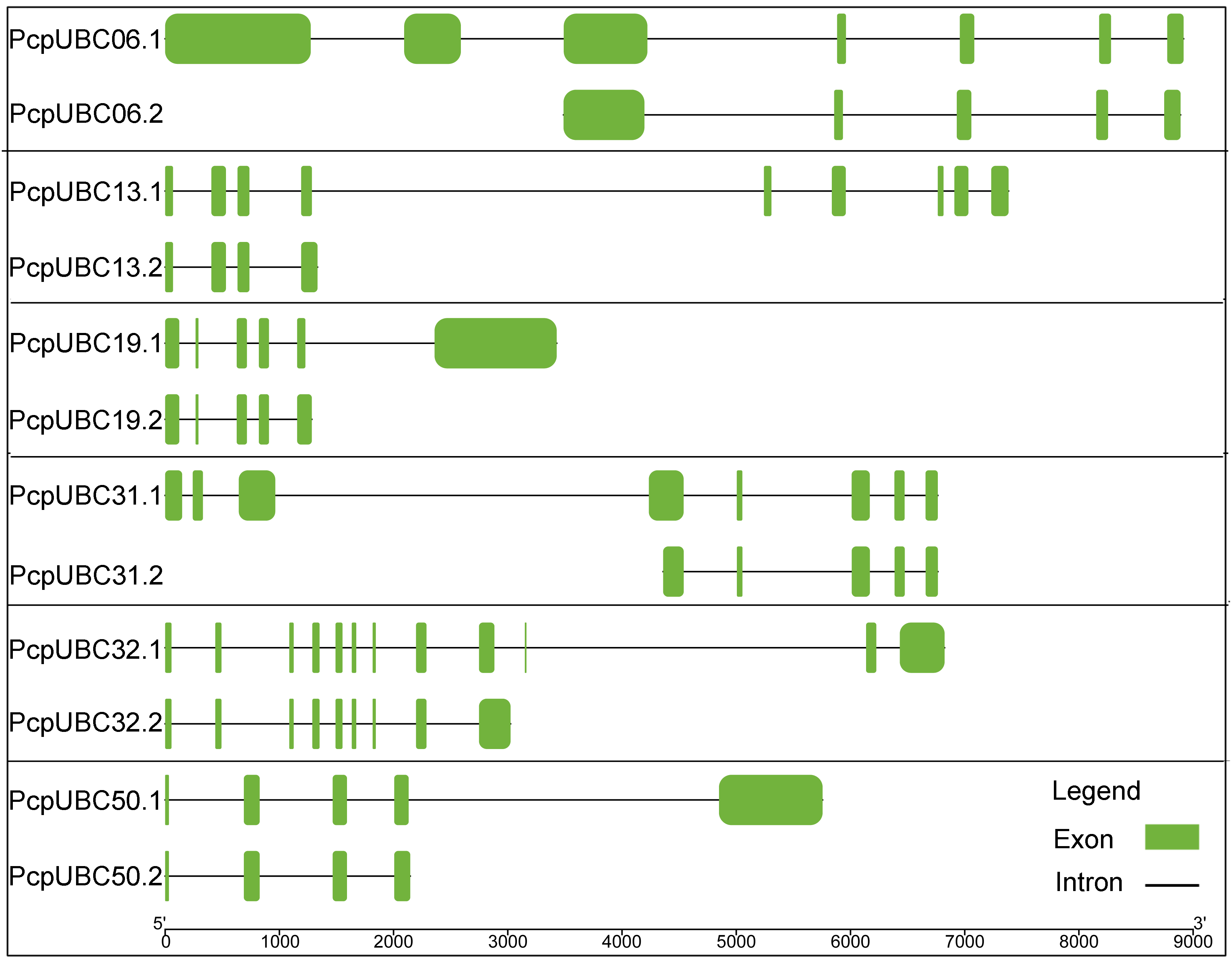

Supplement: Supplementary file 1 [file cells-07-00077-s001.zip › Figure S3.tif]

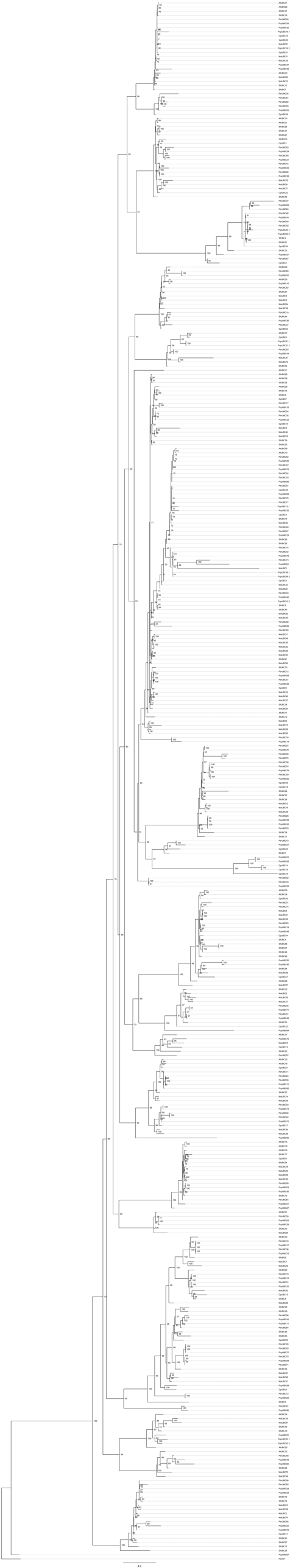

Supplement: Supplementary file 1 [file cells-07-00077-s001.zip › Figure S4.pdf]
